# Incorporation of Brazilian Diatomite in the Synthesis of An MFI Zeolite

**DOI:** 10.3390/molecules24101980

**Published:** 2019-05-23

**Authors:** Paloma Vinaches, Anderson Joel Schwanke, Christian Wittee Lopes, Iane M. S. Souza, Jhonny Villarroel-Rocha, Karim Sapag, Sibele B. C. Pergher

**Affiliations:** 1 Laboratório de Peneiras Moleculares, Universidade Federal do Rio Grande do Norte, 59078-970 Natal, Brazil; souzasiane@gmail.com; 2 Instituto de Tecnología Química (Universitat Politécnica de València – CSIC), 46022 Valencia, Spain; chriswittee@gmail.com; 3 Laboratorio de Sólidos Porosos, Instituto de Física Aplicada, CONICET-Universidad Nacional de San Luis, San Luis C.P. 5700, Argentina; jhoviro@gmail.com (J.V.-R.); sapag@unsl.edu.ar (K.S.)

**Keywords:** diatomite, MFI zeolite, microporous material, alternative sources

## Abstract

The need for greener procedures is a fact to reduce residues, to decrease industrial costs, and to accomplish the environmental agreements. In an attempt to address this question, we propose the addition of a natural resource, Brazilian diatomite, to an MFI zeolite traditional synthesis. We have characterized the resulting product with different techniques, such as X-ray diffraction, microscopy, and gas sorption, and, afterwards, we evaluate the greenness of the process by the Green Star method. The results were promising: We obtained the desired topology in the form of small crystallites aggregated and a pore diameter of 0.8 nm. In conclusion, the product has the necessary characteristics for an adsorption or catalytic future tests and escalation to industrial production.

## 1. Introduction

Zeolites are microporous crystalline solids usually composed of SiO_4_ and AlO_4_ tetrahedra coordinated by oxygen atoms [1]. The organization of these tetrahedral units generates porous structures with different cavities and channels (typically up to 2 nm). They are a class of molecular sieves widely used for adsorption, separation, ion exchange, and catalytic processes [2,3]. The use of zeolites is an alternative solid-acid technology free from the drawbacks of homogeneous catalysts, such as low yields, low recovery, environmental impacts, high investment, and corrosive catalysts [4]. Recently, their use reached other fields—e.g., medicine, cosmetics, food industry, microelectronics, and luminescence [5,6].

**Figure 1:**
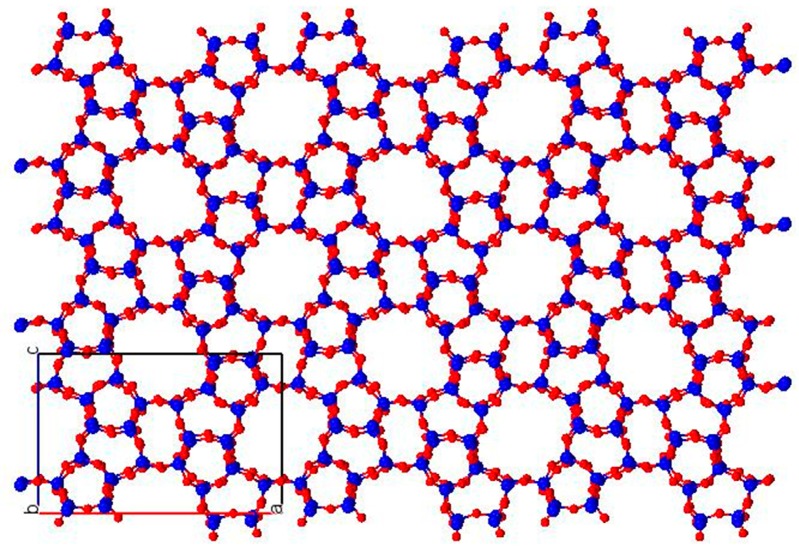
MFI structure along the b-axis, plane (0 -2 0), represented with the program Mercury [11].

According to the International Zeolite Association (IZA), there are more than 200 different zeolitic topologies but only 16 are industrially used [7,8]. ZSM-5 belongs to the MFI topology and is one of the most important zeolites applied as catalyst for alkylation and isomerization reactions in the oil refining industry [9,10]. This zeolite, represented in Figure 1, is composed of ten different natural tilings: *t-bog-1*, *t-kah*, *t-mel*, *t-mel-1*, *t-mel-2*, *t-mfi-1*, *t-mfi-2*, *t-pen*, *t-pes*, and *t-tes*. Its framework density is 18.4 tetrahedra/1000 Å^3^ and its crystal system is orthorhombic.

In the last few years, several efforts have been made to optimize the synthesis of zeolites by using cheaper and/or eco-friendly routes, employing alternative- and low cost-sources of reactants (raw materials or wastes), saving energy by low synthesis temperature and fast crystallization times, aided by microwave and ultrasound procedures and by the recovery and reuse of the mother liquor reactants [12]. Specifically, it was estimated that the cost of reactants for the synthesis of zeolites is ca. 40% [13]. The examples of commercial reactants are silica sources used for the synthesis of zeolites—such as fumed silica, colloidal silica, tetramethylorthosilicate (TMOS), tetraethylorthosilicate (TEOS), silicic acid, and silicon. Some examples of aluminum sources are aluminum nitrate Al(NO_3_)_3_, aluminum sulfate Al_2_(SO_4_)_3_, and aluminum isopropoxide Al(O-i-Pr)_3_. These sources are not considered eco-friendly because they are obtained after several treatments and are associated with the production of a large amount of residues [14].

Among the inorganic reactants considered eco-friendly are raw materials such as clay minerals (kaolin, smectite, sepiolite and palygorskite), rice husk silica, fly coal ash, perlite, and diatomite [13,15,16]. However, in most synthesis procedures using raw materials, the starting material is pre-treated with acid or alkali at high temperatures (>600 °C) to amorphize the previous phase, obtaining SiO_4_ and AlO_4_ available for the nucleation and crystallization of the desired zeolite [14]. Thus, the search for strategies able to be applied in zeolite synthesis incorporating low cost and environmentally friendly raw materials free from pre-treatments is still challenging and attractive.

Among the alternative inorganic sources, diatomites are formed of siliceous fragments from accumulation of microscopic diatomaceous algae carapaces fossilized since the Precambrian period [17]. They are natural porous materials whose properties are light weight, non-toxic, and low cost, with a broad spectrum of applications in industry as filtering agents, fillers for paint and pigments, elastomers, adsorbents for dyes in aqueous solutions, nanocarriers for siRNA transport inside cancer cells, and drug delivery systems [18,19,20,21,22]. There are several articles reporting the use of previously calcined diatomites as silicon source for zeolite synthesis. It was verified that within two hours of crystallization, zeolite A was obtained with 51% crystallinity [23]. Mordenite was studied with and without template in hydrothermal conditions, proposing the possible role of diatomite as a template too [24]. Another study carried out to synthesize zeolite Y reported the use of various impure diatomites previously treated with sulfuric acid, obtaining the zeolite with low Si/Al ratio and comparing its behavior to the colloidal silica used in traditional syntheses [25]. Meanwhile, for ZSM-5, the use of two pretreated diatomitic materials were studied, one with aqua regia (mixture of nitric acid and hydrochloric acid in a molar ratio of 1:3) and the other only with hydrochloric acid, obtaining a synthesis yield of 80% in weight [26].

Considering the increasing interest for greener routes for microporous materials and knowing through previous works the possibility of applying pre-treated diatomites in the synthesis of porous materials, our work was focused on the synthesis of the MFI zeolite (ZSM-5), incorporating on it 33% of a Brazilian diatomite without any pre-treatment step to achieve a greener synthesis route.

## 2. Results and Discussion

**Figure 2:**
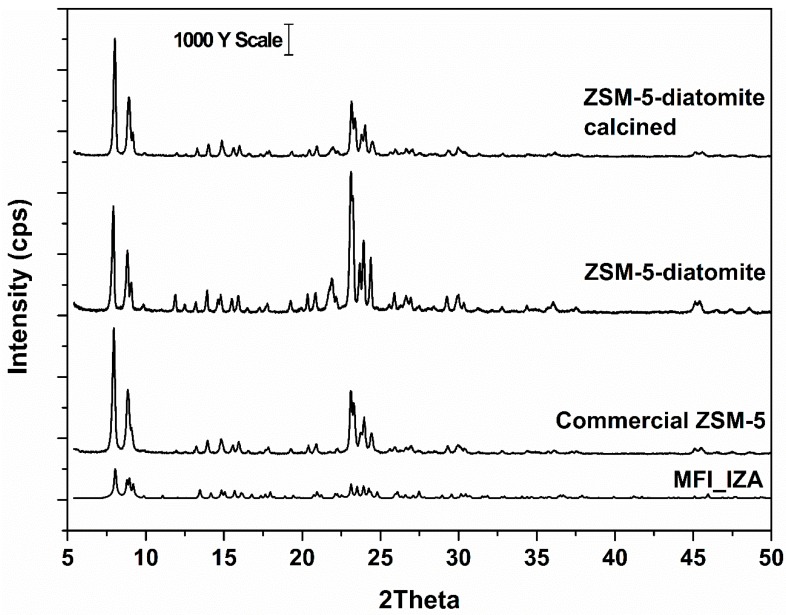
X-ray diffractograms of the zeolitic samples obtained with diatomite showing the success of obtaining MFI zeolitic topology. This result was maintained even after calcination.

X-ray diffractograms (XRD) of the obtained samples, Figure 2, were compared with the commercial ZSM-5 and provided by the IZA database coinciding with the MFI topology, confirming the success of the synthesis procedure [8]. The absence of an amorphous halo related to the diatomite confirms the transformation of this raw material in the final product. Notwithstanding, we found some Bragg reflections related to minor impurity phases. The calcination process maintained the products unchanged (no phase transition), so the next characterizations were focused on the uncalcined sample, except for the sorption experiments that will test the calcined samples.

The Si/Al molar ratio of the uncalcined product was calculated by Inductively Coupled Plasma-Optical Emission Spectrometry (ICP-OES), resulting in a value of 16.2. We managed to synthesize an Al-rich material due to the combination of the traditional and the alternative sources, as the diatomite presented a Si/Al molar ratio of 28.8 also calculated by ICP.

**Figure 3:**
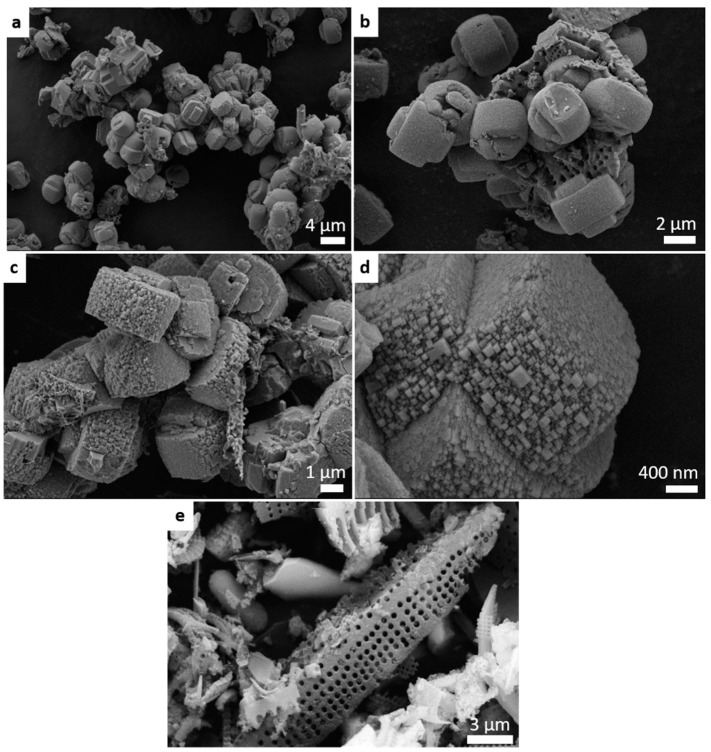
SEM micrographs of the zeolitic product ZSM-5-diatomite observed at several magnifications (**a**–**d**), and of the initial raw material, diatomite (**e**).

Figure 3 shows the scanning electron microscopy (SEM) micrographs of the zeolitic product ZSM-5-diatomite (a–d) and the initial diatomite (e). At small magnification values, we identified aggregates of coffin-shape like morphology particles with sizes of ca. 4.0 × 3.0 µm forming the zeolitic product (a and b). However, observed closely, at higher magnifications, the apparently smooth surfaces revealed to be agglomerates of nanozeolites (c and d). Their sizes are around 57 nm. This type of agglomerates was already reported for the traditional synthesis [27], being another proof of the similarity of the products that is important for the future industrial use of this alternative methodology. The appearance of a porous surface (d) similar to the diatomite employed (e) indicated that the raw material was not completely consumed in the synthesis. Some intergrowth zeolite-diatomite also occurred (c).

**Figure 4:**
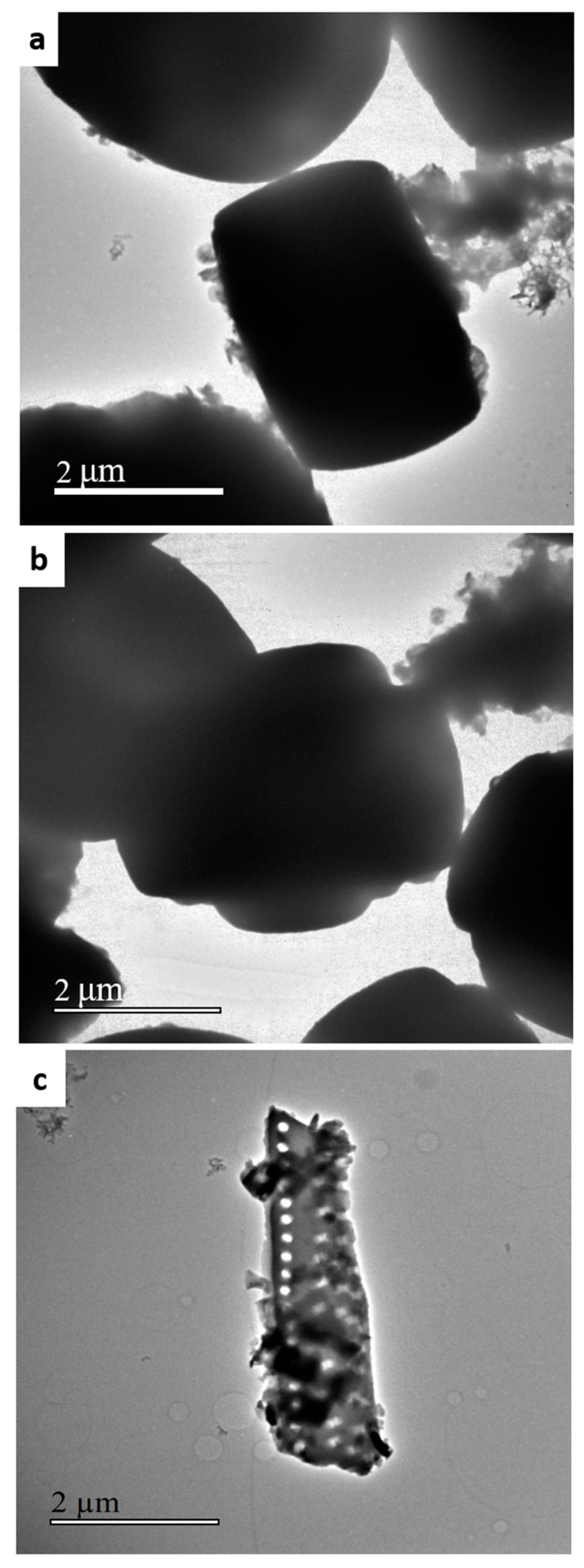
TEM micrographs of the zeolitic product ZSM-5-diatomite observed by different perspectives (**a**,**b**), and also showing the intergrowth zeolite-diatomite (**c**).

Transmission electron microscopy (TEM) micrographs of the zeolitic material are shown in Figure 4. We observed a single aggregate of nanozeolites (a and b), confirming the size calculated in the SEM micrographs. We managed to characterize with this technique an intergrowth showing an intact particle of the diatomite carapace with dimensions of 5.0 × 1.2 µm and macropores with diameters of 120 nm, also showing some smaller zeolites on its surface. This fact indicates that larger diatomitic particles are more difficult to dissolve.

**Figure 5:**
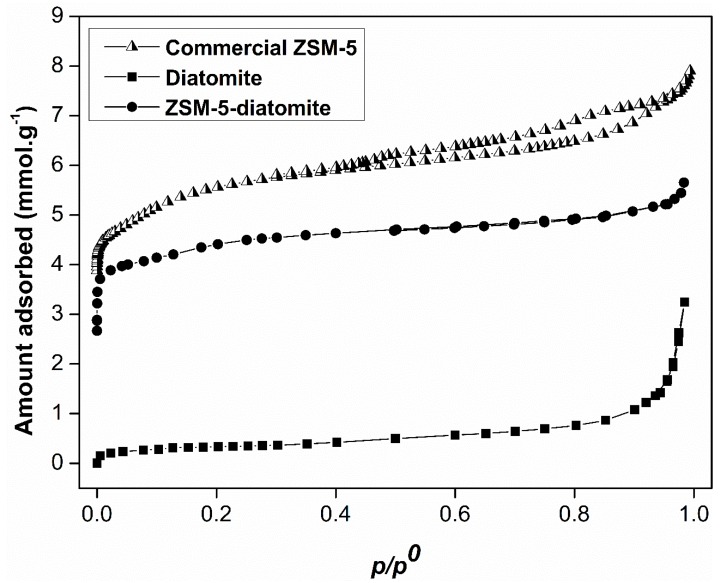
Nitrogen adsorption/desorption isotherms for the diatomite, the commercial ZSM-5 and the synthesized ZSM-5-diatomite samples. The raw material presented a Type II isotherm, meanwhile the zeolites showed Type I isotherms.

A sorption systematic study of the raw material, diatomite, in comparison with the zeolitic products, ZSM-5-diatomite and commercial ZSM-5 was performed. Starting with the nitrogen adsorption-desorption isotherms at 77 K, the results are shown in Figure 5. According to the IUPAC classification [28], the diatomite exhibited a Type II isotherm, characteristic of non-porous or macroporous materials. Meanwhile, the ZSM-5-diatomite presented a Type I isotherm at low relative pressure, which is indicative of the microporous nature of this sample. This same behavior was also observed in the isotherm of the commercial ZSM-5. In addition, the isotherm of the diatomite showed a rapid increase of the nitrogen adsorbed amount at high relative pressure (near to 1) indicating the presence of larger mesopores or narrow macropores. The specific surface area (*S_BET_*) was estimated by BET (Brunauer, Emmett and Teller) method [29,30] from the nitrogen adsorption isotherm data applying the methodology proposed by Rouquerol et al., resulting in 357 m^2^ g^−1^ for the ZSM-5-diatomite and 440 m^2^ g^−1^ for the commercial ZSM-5. This difference might be a consequence of the impurities and presented in ZSM-5-diatomite.

**Figure 6:**
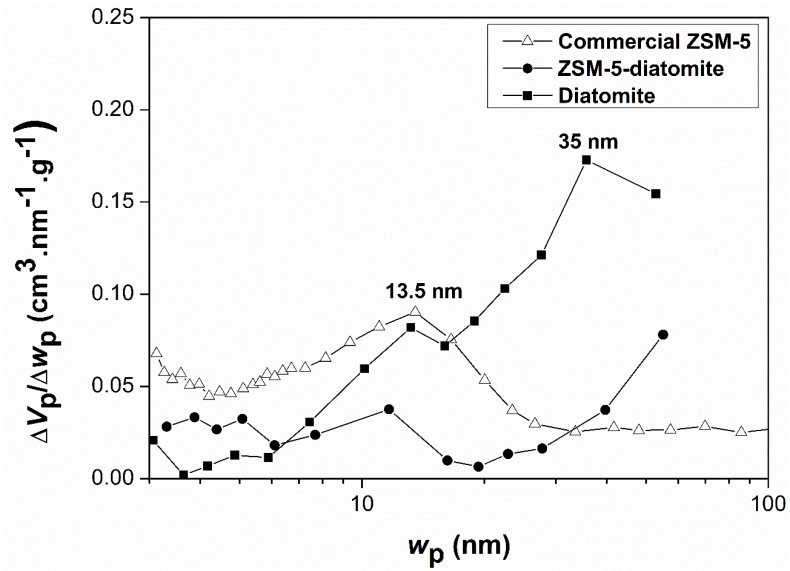
Mesopore-size distribution of the different materials calculated by the Villarroel-Barrera-Sapag (VBS) method using the adsorption branch data of the nitrogen isotherm.

The mesopore-size distributions from the nitrogen sorption experiments were determined applying the Barrett-Joyner-Halenda (BJH) method (which assume cylindrical pores), using the adsorption branch data and considering the correction factor proposed in the Villarroel-Barrera-Sapag (VBS) method (Figure 6) [31]. The results revealed that the raw diatomite presented pores higher than 20 nm; while the ZSM-5-diatomite, as for the commercial ZSM-5, also presented mesopores, distributed between 5 and 55 nm. Comparing the mesopore sizes of these zeolites, ZSM-5-diatomite present mesopores larger than the commercial ZSM-5, with modal pore sizes of 35 nm and 13.5 nm, respectively. These initial results provided a guideline for further complementary analysis with more suitable probe molecules—i.e., CO_2_ adsorption for the zeolitic ZSM-5-diatomite product (for the study of narrow micropores) and Hg intrusion porosimetry for the raw material (for the study of macropores).

**Figure 7:**
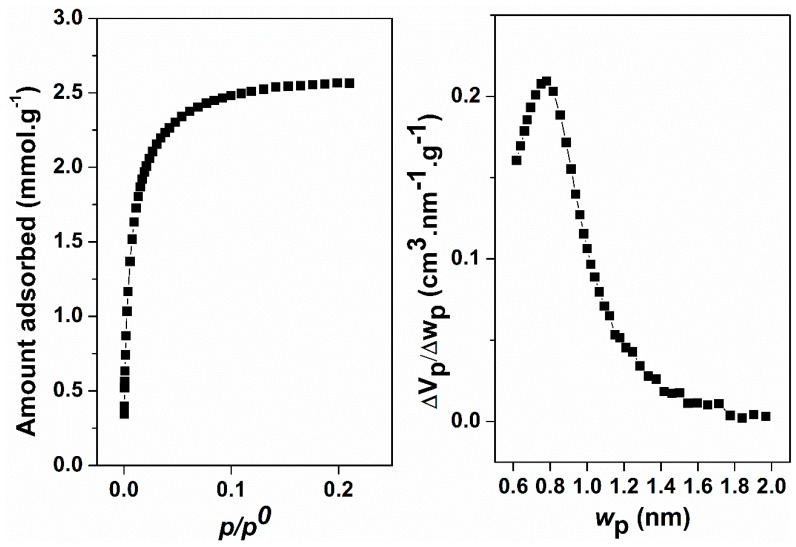
CO_2_ adsorption isotherm (left) for the zeolitic sample, ZSM-5-diatomite, and the corresponding micropore-size distribution (right), resulting approximately a modal micropore size of 0.8 nm.

The CO_2_ adsorption isotherm at 273 K of the zeolitic product (Figure 7, left) was classified as a Langmuir-type isotherm [28,29,30] corresponding Type I in IUPAC classification, evidencing the presence of narrow micropores. The micropore-size distribution (Figure 7, right) obtained using the HK (Horváth-Kawazoe) method for cylindrical pores [30] confirmed the previous evidence and reported a pore size of approximately 0.8 nm. This value is slightly higher than expected [8], which may be related to the effect of the agglomeration of small crystals. In addition, the micropore volume was calculated by the DR (Dubinin-Radushkevich) methodology applied to CO_2_ adsorption data [32], resulting in 0.13 cm^3^ g^−1^.

**Figure 8:**
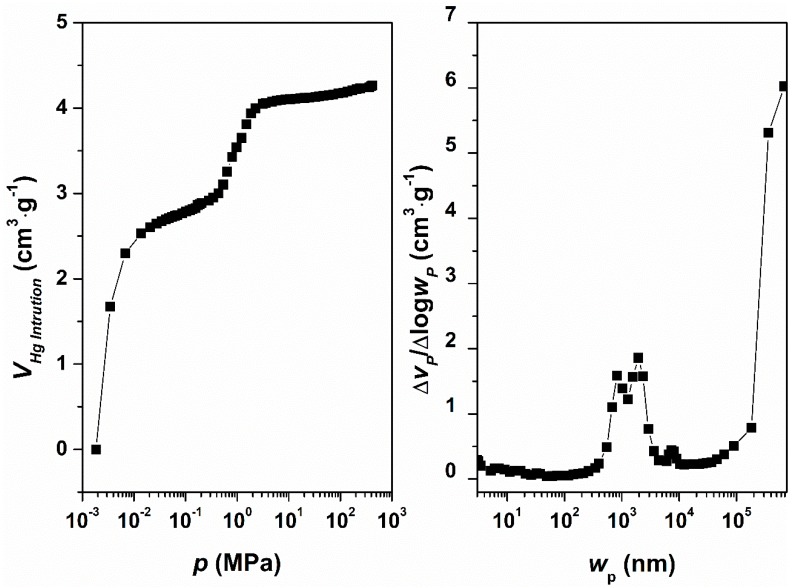
Hg intrusion porosimetry curve (left) for the diatomite and the corresponding macropore-size distribution (right), resulting in smaller-size macropores around 850–2000 nm and bigger than 400 µm.

The microporous zeolite was the result of the conversion of a macroporous material, as observed previously. The Hg intrusion curve (Figure 8, left) confirmed this statement, calculating a macropore volume of 4.2 cm^3^ g^−1^. The macropore-size distribution (Figure 8, right) was divided into two main zones: Smaller-size macropores between 850 and 2000 nm, and bigger-size macropores of >400 µm (due to the interparticle space), in agreement with the SEM and TEM observations.

Finally, we evaluated the greenness of the zeolite synthetic process, applying the semi-quantitative Green star methodology as described by Ribeiro et al. [33] This evaluation considers the twelve principles of green chemistry, named: Prevention (P1), atom economy (P2), less hazardous chemical synthesis (P3), designing safer chemicals (P4), safer solvents and auxiliary substances (P5), increase energy efficiency (P6), use renewable feedstocks (P7), reduce derivatives (P8), catalysts (P9), design for degradation (P10), real-time analysis for pollution prevention (P11), and safer chemistry for accident prevention (P12). Even though they are twelve different aspects, not all are applied in every case. An example is the P9, only considered in case of catalyst involved in the reaction. Scores (S) from 1 to 3 are assigned to the applicable parameters, with 3 being the “greenest” value. A more detailed description on how to calculate the scores is described elsewhere [33]. Once the scores are obtained, they are represented in a radar chart (1 parameter per vertex following an increasing numerical order, joining them by straight lines) and the result will give an idea of the greenness by the filling of the chart (the fuller, the greener the process will be). So, for the present research work, the applicable parameters are P1–P3, P5–P9, and P12. Then, the scores for the evaluated parameters resulted as presented in Table 1.

**Figure 9:**
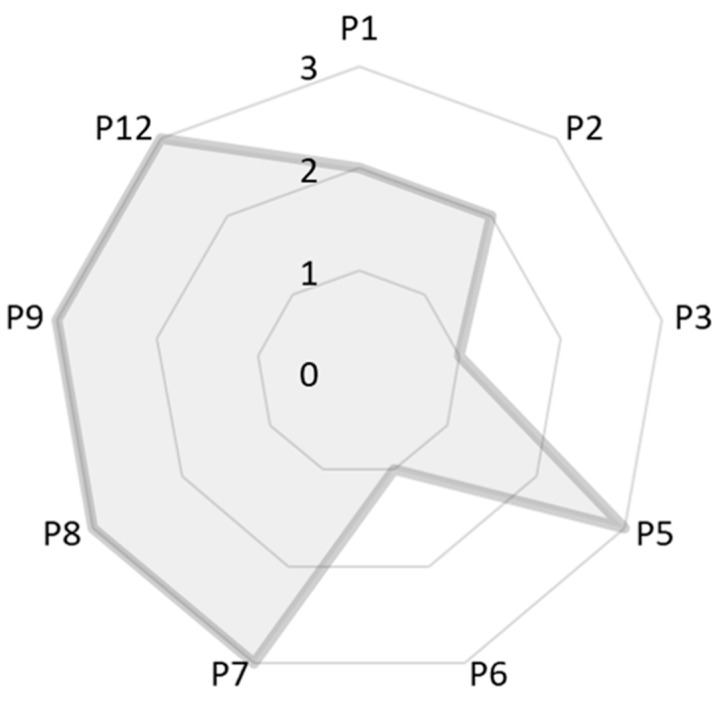
Green Star chart for the synthesis of ZSM-5 adding Brazilian diatomite as raw material.

The twelve principles of Green Chemistry must be followed (all parameters need to have a score equal to 3) to be considered just “green” or “not”, but not all the synthetic areas can apply them all. The Green Star methodology helps in this evaluation giving an idea of the “greenness” parameter of a synthesis, and it was described as a semi-quantitative analysis. Then, further analysis of these results and a coherent application to each area were needed. This type of analysis was not applied in zeolite synthesis before, as far as we know. Then, we need to establish a system to discuss this punctuation. The nine parameters applied should have a value of 3 to be considered “fully green”, but as Green Chemistry for zeolite synthesis is still in development, we consider 50% as a good percentage of “greenness” to start with. For the present research work, we represented these results as recommended by the methodology (Figure 9), and two thirds of the chart were completely full (>50% of the chart). So, this synthesis can be considered as part of the Green Chemistry for zeolite synthesis. But, this also means that there is still margin to improve.

## 3. Materials and Methods

The commercial zeolite was marketed by Zeolyst (Conshohocken, PA, US). The synthesis of the ZSM-5-diatomite zeolite was based on the methodologies reported by Van Grieken et al. and Song et al. [27,34]. Briefly, 1.075 g tetrapropylammonium hydroxide (TPAOH, 1 M in water, Sigma-Aldrich, St. Louis, MO, USA), 0.119 g aluminum isopropoxide (TIPAl, 98%, Merck, Darmstadt, Germany), 0.090 g sodium hydroxide (NaOH, 98%, Sigma-Aldrich, St. Louis, MO, USA), and 3.609 g distilled water were weighted separately and mixed in a polypropylene vessel. The mixture was magnetically stirred in a water bath with ice until dissolution was complete. At that moment, 3.011 g (TEOS, Sigma-Aldrich, 98%) was added and left stirred overnight to ensure the TIPAl and TEOS hydrolysis. Then, 1 g diatomite (composition: 91.8% SiO_2_, 5.8% Al_2_O_3_, 1.3% Fe_2_O_3_, 0.5% CaO, 0.5 K_2_O, 0.1% others; simplifying the calculi to 3 (traditional source): 1 (alternative material)) was added and magnetically stirred for 1 h. Finally, the synthesis gel was divided among the Teflon autoclaves and introduced in stainless steel autoclaves. The synthesis was performed under static conditions at 160 °C for 5 d. After this time, the samples were filtered out, washed with distilled water, and dried. The samples were calcined at 550 °C for 6 h with a heating ramp of 2 °C/min (based on our previous experience with MFI zeolite [35]) for comparison by XRD.

The resulting zeolite was characterized by XRD, ICP-OES, SEM, TEM, and various sorption techniques, such as nitrogen and carbon dioxide sorption and mercury porosimetry.

The X-ray diffractograms were obtained in a Bruker D2 Phaser equipment with a Lynxeye detector and using Cu Kα radiation (Bruker, Billerica, MA, USA). The divergent slit employed had a 0.6 mm opening and the central slit left a 1 mm opening. The measuring step chosen was 0.02° and the acquisition time 0.1 s.

The morphology of the sample was studied by FE-SEM (field emission SEM) using a ZEISS Ultra-55 microscope (Zeiss, Oberkochen, Germany). The powder sample was deposited in double-sided carbon tape and analyzed without metal covering. The image of Figure 4e was obtained with a FE-SEM-XL30 Philips microscope (FEI, Hillsboro, Oregon, USA) and the sample were coated with a gold layer.

The zeolitic sample was also studied by electron microscopy in a JEOL-JEM-2100F microscope operating at 200 kV in transmission mode (TEM, JEOL, Tokyo, Japan). Prior to microscopy analysis, the sample was suspended in isopropanol and submitted to ultrasonication for approximately one minute. Afterwards, the suspension was allowed to slowly decant, and a drop was extracted from the top side and placed on a carbon-coated nickel grid.

The chemical analysis of the studied materials was carried out by ICP-OES in a Varian 715-ES. The powder samples (approx. 20–30 mg) were dissolved in an acid mixture of 20% HNO_3_:20% HF:60% HCl (% volume).

The nitrogen adsorption/desorption isotherms at −196 °C were obtained in a manometric adsorption equipment (Autosorb-1MP, from Quantachrome Instruments, Boynton Beach, FL, USA). The CO_2_ adsorption isotherm at 0 °C up to 1000 kPa was performed in an ASAP 2050 analyzer (from Micromeritics, Norcross, GA, USA). The samples were previously degassed at 80 °C for 24 h, reaching a final pressure of 0.5 Pa. The Hg intrusion porosimetry was carried out in an Autopore III equipment (from Micromeritics, Norcross, GA, USA), from 0.0034 MPa up to 414 MPa of pressure.

## 4. Conclusions

In this research work, we studied the incorporation of a natural raw material, a Brazilian diatomite, in the synthesis of an industrially relevant zeolite, namely ZSM-5. The crystalline ZSM-5 product obtained was similar to the classical material reported in the literature, coinciding the Bragg reflections and even imitating a well-known morphology. The small crystal agglomerates had an unexpected influence on the sorption experiments, observing a widening of the medium pore size instead of finding mesoporosity. We also observed that the final material had a higher Al content that initially previewed, explained with the combination of raw materials (traditional and alternative). By applying a relative new concept (Green Star methodology), a final evaluation on the greenness let us conclude that this synthesis can be included among the green chemical procedures. To sum up, this research work presented a methodology to lower industrial costs of the ZSM-5 production by the introduction of a natural material, diatomite, in its synthesis.

## Figures and Tables

**Table 1:** Green Star evaluation.

Parameter	Score	Comments
P1	2	This score implies that the waste generated involved a moderate risk to human health and environment. Even though we did not study the reuse and recycle of the mother liquors in this research work, it is possible to perform it as demonstrated elsewhere. This fact reduced this score to the former value of 2.
P2	2	We considered the mother liquors as by-products.
P3	1	The presence of tetraethylorthosilicate and sodium hydroxide decreased the score. To perform basic media synthesis of zeolite, it is compulsory to use a hydroxide source, so the score cannot be higher in the obtention of zeolites.
P4	-	The resulting product was not planned for a biological use.
P5	3	It is a consequence of choosing distilled water as solvent.
P6	1	The synthesis was performed at a temperature higher than 100 °C. Most zeolitic topologies are obtained at this temperature or superior, which means that further study is still needed in this sense.
P7	3	This synthesis was performed incorporating diatomite, a natural raw material.
P8	3	No derivatization was needed.
P9	3	Not using any catalyst for the synthesis.
P10	-	Zeolites are also natural materials.
P11	-	There was no formation of hazardous substances during or after the synthesis.
P12	3	The hydrothermal synthesis in basic media is one of the most well-known and most controlled among the zeolite synthesis methods.
